# Impact of Pneumococcal Conjugate Vaccine on Vaccine Serotype–Specific Pneumonia

**DOI:** 10.1093/cid/ciaa1867

**Published:** 2020-12-18

**Authors:** Keith P Klugman, Gail L Rodgers

**Affiliations:** Bill and Melinda Gates Foundation, Seattle, Washington, USA

**(See the Major Article by Lewnard et al on pages e1423–33.)**

Keywords. *Streptococcus pneumoniae*; pneumococcus; serotype; pneumonia; pneumococcal conjugate vaccine.

A range of 20–40% efficacy of pneumococcal conjugate vaccine (PCV) in preventing chest radiograph–confirmed pneumonia in children has been established since the early development of a World Health Organization (WHO)–endorsed definition of alveolar pneumonia [1], which was used prospectively to define pneumonia in 2 large randomized trials of PCV9 in Africa [2, 3]. A retrospective analysis confirmed a similar impact of PCV7 using this definition in a clinical trial of PCV7 in Northern California [4, 5], but no impact was detected for unclear reasons using that vaccine among the Navajo and White Mountain Apache communities [6]. An investigational PCV11 provided similar impact on alveolar pneumonia in a clinical trial in the Philippines [7] and PCV10 in clinical trials in Finland [8] and Central and Latin America [9].

A limitation of all these trials is that the serotype distribution of pneumococci causing pneumonia is essential to understanding trial results. Yet, beyond the identification of a small fraction of cases by blood culture in these trials [2–8] and by lung puncture in a few cases in The Gambia [3], there has been no way to estimate serotype-specific vaccine efficacy against chest radiograph–confirmed pneumonia.

Vaccine studies comparing pneumonia incidence in observational studies pre– and post–PCV introduction have demonstrated remarkable effectiveness globally and have likewise been frustrated by a lack of a measure to define direct vaccine impact on the pneumococcal serotypes causing pneumonia.

Although estimates of the contribution of the pneumococcus to all cases of alveolar pneumonia are controversial, the impact of PCV in a range of 20–40% reduction in all-cause alveolar pneumonia, when the PCV serotypes only comprise 50–70% of the pneumococcal serotypes causing the disease (serotype distribution inferred from the blood culture–positive cases), suggests that even if the pneumococcus is responsible for as much as 60% of all chest radiograph–confirmed alveolar pneumonia, the efficacy of the vaccine in preventing the fraction attributable to the vaccine serotypes must be high.

The only estimate to date of serotype-specific PCV impact on alveolar pneumonia was reported in adults during a PCV13 trial in which cases had a serotype-specific urinary assay performed [10]. This study found a serotype-specific efficacy of 46%, but efficacy of PCV wanes in the elderly and thus the efficacy in children may be higher. Unfortunately, the serotype-specific urinary assay used in that study has not yet been validated for use in children, in whom high-density nasopharyngeal (NP) carriage in asymptomatic children may confound results, requiring different cutoffs of the assay to define serotype-specific pneumonia.

In this issue of *Clinical Infectious Diseases*, Lewnard and colleagues [11] have proposed an analysis to assess PCVs’ serotype-specific pneumonia effectiveness based on the idea of progression of NP carriage to disease. Pneumococci are naturally carried in the nasopharynx in up to 90% of asymptomatic children, thus frustrating attempts to use NP carriage to define the etiological role of these bacteria. It is, however, well known that children who have pneumococcal pneumonia carry the causative bacterium in the nasopharynx at a high frequency, at least prior to antibiotic administration. Therefore, the authors have presented the hypothesis that the ratio of the carriage of vaccine serotype–specific pneumococci in the nasopharynx of vaccinated children who have progressed to pneumonia will be less (compared with vaccinated control children without pneumonia) than the ratio of vaccine serotypes among unvaccinated children with pneumonia (compared with unvaccinated control children without pneumonia) and that one minus the difference in these ratios can be computed to a measure of vaccine efficacy against vaccine serotype–associated pneumococcal pneumonia. This is a complex analysis as the odds ratios are calculated using multivariable models adjusted for difference in covariates such as age, seasonality, and other differences between cases and controls.

Despite the systematic collection over many years of serotype-specific carriage from thousands of cases and controls, the power of their analysis to determine significant differences in these ratios is limited and the only point estimates with confidence intervals not crossing zero in their adjusted analysis were for children 12 to 36 months of age following receipt of a booster dose of PCV13 at 12 months of age. Nonetheless, the point estimates of vaccine effectiveness in this group were in excess of 80%, which suggests a level of effectiveness similar to that against serotype-specific invasive disease. As mentioned above, these high estimates of effectiveness against vaccine serotype–associated alveolar pneumonia are consistent with the multiple observations of an overall 25% efficacy of the vaccine against all alveolar pneumonia. An 80% effectiveness in this study implies that the vaccine serotypes caused a mean of 31% of the alveolar pneumonias in those studies and at PCV’s 70% coverage of disease-causing serotypes, that the pneumococcus overall was responsible for at least 44% of those WHO-defined pneumonias.

The role of the pneumococcus in severe pneumonia in hospitalized children is best measured by these types of probe studies, which lead to the consistent calculation that the pneumococcus is responsible for significant morbidity and mortality. In low- and middle-income countries, the serotype distribution may be more diverse, suggesting that an even larger fraction of alveolar pneumonia may be vaccine preventable by next-generation PCVs, comprising 20 or more serotypes.

Ultimately, a direct diagnostic approach to the serotype-specific detection of pneumococcal pneumonia in children remains elusive and, while these data suggest that vaccine effectiveness may be high, the study, despite its size, was underpowered to answer questions about serotype-specific disease. The further development and evaluation of a serotype-specific urinary antigen assay in children thus remain an urgent priority.

## Note

***Potential conflicts of interest.*** The authors: No reported conflicts of interest. Both authors have submitted the ICMJE Form for Disclosure of Potential Conflicts of Interest. Conflicts that the editors consider relevant to the content of the manuscript have been disclosed.
